# Douchi (fermented *Glycine max* Merr.) alleviates atopic dermatitis-like skin lesions in NC/Nga mice by regulation of PKC and IL-4

**DOI:** 10.1186/s12906-016-1394-4

**Published:** 2016-10-24

**Authors:** A-Ram Jung, Sang-hyun Ahn, In-Sik Park, Sun-Young Park, Seung-Il Jeong, Jin-Hong Cheon, Kibong Kim

**Affiliations:** 1 Department of Korean Pediatrics, Hospital of Korean Medicine, Pusan National University, 20 Geumo-ro, Mulgeum-eup, Yangsan-si, Gyeongsangnam-do 50612 Republic of Korea; 2 Department of Anatomy, College of Korean Medicine, Semyung University, 65 Semyung-ro, Jecheon-si, Chungcheongbuk-do 27136 Republic of Korea; 3 Department of Anatomy, College of Korean Medicine, Dongguk University, 123 Dongdae-ro, Gyeongju, Gyeongsangbuk-do 38066 Republic of Korea; 4 Department of Physiology, College of Korean Medicine, Semyung University, 65 Semyung-ro, Jecheon-si, Chungcheongbuk-do 27136 Republic of Korea; 5 Jeonju AgroBio-Materials Institute, Jeonju, Jeollabuk-do 54810 Republic of Korea; 6 Department of Korean Pediatrics, School of Korean Medicine, Pusan National University, 49 Pusandaehak-ro, Mulgeum-eup, Yangsan-si, Gyeongsangnam-do 50612 Republic of Korea

**Keywords:** Douchi, Fermented soybean, Atopic dermatitis, Th2 cytokine, PKC

## Abstract

**Background:**

Douchi (fermented *Glycine max* Merr.) is produced from fermented soybeans, which is widely used in traditional herbal medicine. In this study, we investigated whether Douchi attenuates protein kinase C (PKC) and interleukin (IL)-4 response and cutaneous inflammation in Atopic dermatitis (AD)-like NC/Nga mice.

**Methods:**

To induce AD-like skin lesions, *D. farinae* antigen was applied to the dorsal skin of 3-week-old NC/Nga mice. After inducing AD, Douchi extract was administered 20 mg/kg daily for 3 weeks to the Douchi-treated mice group. We identified the changes of skin barrier and Th2 differentiation through PKC and IL-4 by immunohistochemistry.

**Results:**

Douchi treatment of NC/Nga mice significantly reduced clinical scores (*p* < 0.01) and histological features. The levels of PKC and IL-4 were significantly reduced in the Douchi-treated group (*p* < 0.01). The reduction of IL-4 and PKC led to decrease of inflammatory factors such as substance P, inducible nitric oxide synthase (iNOS) and Matrix metallopeptidase 9 (MMP-9) (all *p* < 0.01). Douchi also down-regulated Th1 markers (IL-12, TNF-α) as well as Th2 markers (IL-4, p-IκB) (*p* < 0.01).

**Conclusion:**

Douchi alleviates AD-like skin lesions through suppressing of PKC and IL-4. These results also lead to diminish levels of substance P, iNOS and MMP-9 in skin lesions. Therefore, Douchi may have potential applications for the prevention and treatment of AD.

## Background

Atopic dermatitis (AD) is a chronic inflammatory skin disease with a complex etiology encompassing immunologic response [1]. Various factors including genetic, environmental, and immunological disruption contribute to skin barrier dysfunction [1]. AD can induce an atopic march, which is a complex process involving sinusitis, allergic rhinitis and asthma. Thus, early treatment is necessary for patients with AD [2].

**Table 1:** Contents of isoflavones from extracts by HPLC-DAD

Sample ID	Contents of isoflavones (μg/mL)			
	daidzin	daidzein	genistin	genistein
Raw soybean	706.41	24.88	937.94	29.19
Douchi	1178.05	170.90	2027.54	265.33

One of the main feature of AD is known to imbalance of Th1/Th2 immune response through the induction of T helper (Th) 2 cytokines such as interleukin (IL)-4 in skin lesions [3]. Recently, several studies have reported that Th2 cytokine activity could control the onset of AD in animal models [4, 5]. Activated Th2 cells secret IL-4, IL-5 and IL-13; IL-4, in particular, has been demonstrated to enhance AD through the promotion of T cell differentiation [6]. IL-4 stimulates the degranulation of mast cells, the release of substance P (which is related to itching) [7], MMP-9 (which induces edema) [8], and iNOS (which is related to angiogenesis) [9]; thereby amplifying inflammatory skin damage [10].

Another primary event in the pathogenesis of AD is the dysfunction of lipid enriched extra-cellular matrix including ceramide in the stratum corneum [11]. The damage of lipid barrier is induced by activation of protein kinase C (PKC), which plays a role as the elicitation of AD [12]. In addition, PKC is crucial for Th2 cytokine activation, which shifts Th1/2 balance to Th2-dominant state, resulting in an acute inflammatory state [13]. Furthermore, this state activates mast cell, which leads to release various inflammatory mediators. This entire process plays a key role in the initiation of AD. Therefore, the suppression of Th2 cytokines by blocking PKC activation is a novel strategy for protecting against AD [14].

With Conventional medicine, AD occurs due to a complicated set of factors. It is characterized by skin barrier dysfunction and the disruption of the immune system involving the network of Th2 cytokines [1]. AD occurs in 23.6 % of infants less than 24 months old in Korea [12]. As a previous study, infants are characterized by Th2-dominant state, therefore when they are exposed to external antigens, infants are more likely to get AD [15]. At present, the typical treatments available are dominated by the corticosteroid medication which has an effect of suppressing Th2 cytokines. However, these steroids can cause adverse effects including a risk of fracture, atrophoderma, and growth delay. Therefore, the development of natural remedies is highly desirable [16].

Within traditional medicine, inflammatory disease is commonly diagnosed as heat syndrome [17]. Heat syndrome (Zheng) is a basic concept in CAM, which may can be represented as symptoms like erythema, flushed face, high fever, red tongue and so on [18]. Heat syndrome is one of major signs of AD in CAM [17], and generally corresponds to inflammation and infection in neuro-endocrine immune system of western medicine [19]. Therefore, CAM treats AD with ‘heat-clearing’ medicines to reduce accumulated heat in skin [20].

Douchi (fermented *Glycine max* Merr.) is a kind of fermented soybean and a representative herbal medicine for reducing ‘heat’ in traditional medicine [21]. Douchi is used as a complementary and alternative medicine in the treatment of heat-related disease such as heartburn, inflammation and common cold [21]. It was found that it contains isoflavones such as genistein, daidzein [22], and its components are known to have an effect of regulating PKC and Th2 response in inflammatory disorders [23–25]. Several animal studies also reported that fermented soybeans, which is similar to Douchi, have therapeutic effects on inflammatory allergic diseases that involve Th2 immunomodulatory activity [26, 27]. As a type of fermented soybean, Douchi also could be expected to have similar effect in inflammation response such as AD.

Inflammation response is closely related to oxidative stress and lipid metabolism [28]. As previous *in vitro* studies, Douchi is known to have anti-oxidative effect and anti-lipid peroxidative effect [29, 30]. Inducible nitric oxide synthase (iNOS) is common essential players of inflammation response and oxidative stress [31], in addition, activation of lipid metabolism increased inflammation reactions by producing pro-inflammatory cytokines [28]. Therefore, the antioxidant and anti-lipid peroxidative property of Douchi implied that it might have possibility of inhibiting inflammation response. However, the role of Douchi on inflammatory disease in skin has not been studied yet.

Thus, based on the known effect of Douchi, we explored to determine whether Douchi alleviates clinical AD symptoms through regulating further expanding inflammatory factors including mast cells, substance P, iNOS and MMP-9 as well as PKC and IL-4. Moreover, we also measured Th1 and Th2 differentiation through checking IL-12, TNF-α (Th1 markers) versus IL-4, p-IκB (Th2 markers) to identify Th1/Th2 imbalance.

## Methods

### Preparation of Douchi extract

The Douchi used in this study was purchased from Namyoung Pham (Muju, Korea). The procedure used to manufacture Douchi was as follows: first, black soybeans were fermented in *Artemisia Apiacea Herba* and *Mori Folium* extracts (1: 1) for 5 days at 37–38 °C; next, 100 g of fermented soybeans were decocted with 1000 mL of distilled water for 3 h and then filtered; after concentrating this mixture to 50 mL under reduced pressure using a rotary evaporator, the filtrate was freeze-dried. We obtained 14.74 g of the extract (yield: 14.74 %) for use.

### Fingerprinting analysis of Douchi extract with high-performance liquid chromatography (HPLC)

HPLC-based fingerprinting was performed using the Agilent technologies 1200 series (Agilent Technologies, CA, USA), a binary solvent delivery pump (G1312A), a vacuum degasser (G1322A), an auto-sampler (G1329A), a diode array detector (G1315D) with detection at 260 nm, thermostate column compartment (G1316A) maintained at 35 °C and AgilentChem Station software. For analytical scale study, concentrations of standard samples and plant extracts were injected (15 μL) onto a AegisPak-LC18 column (4.6 × 150 mm; pore size, 3 μm). The mobile phases were solvent A [0.1 % formic acid aqueous (v/v)] and solvent B (acetonitrile) at a flow rate of 0.5 mL/min. The gradient flow was as follows: initiation-5 % B, 20 min-20 % B, 28 min-30 % B, 32 min-60 % B, 37 min-60 % B, 40 min-5 % B. A standard solution, containing daidzin (1), daidzein (2), genistin (3) and genistein (Sigma-Aldrich, St. Louis, MO, USA), was prepared by dissolving in distilled water (10 mg/100 mL). The solution was filtered through a 0.45 μm membrane filter and HPLC was performed. The concentrations of four compounds in the exact were calculated with reference to standard curve of the corresponding compound.

**Fig. 1:**
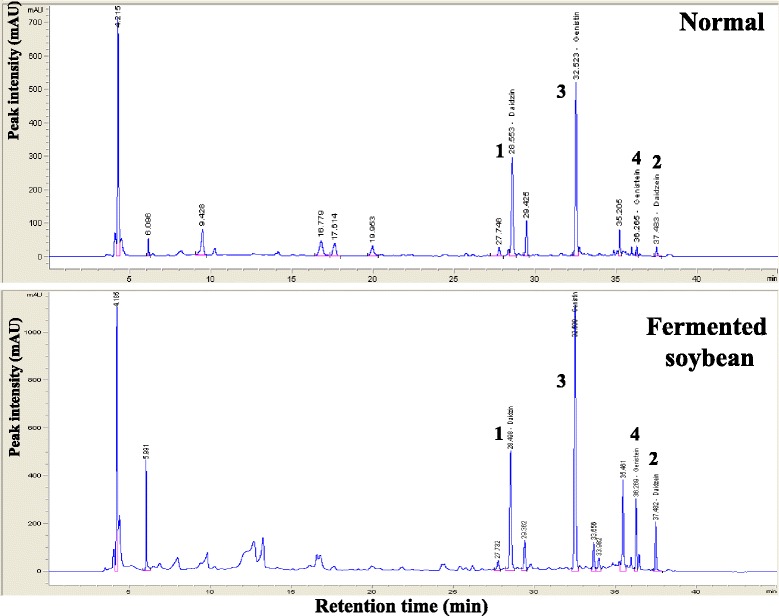
HPLC-DAD analysis of the exacted Douchi (fermented *Glycine max* Merr.). Raw soy bean (Normal) and Douchi (Fermented soybean) analyzed by HPLC. Peak number : daidzin (1), daidzein (2), genistin (3) and genistein (4) at 260 nm

As shown in Fig. 1, daidzin (1), daidzein (2), genistin (3) and genistein (4) were the major compounds of Douchi and raw soybean extract, which detected amounts of 1178.0, 170.9, 2027.5 and 265.3 μg/mL of the Douchi extract, respectively. In raw soybean samples, the amount of individual isoflavones was found to be 706.4, 24.8, 937.9 and 29.2 μg/mL (Table 1). As anticipated, the overall chemical content in raw soybean was significantly lower than that in Douchi. It was no doubt that the proportion of genistin (2027.5 μg/mL) was highest in Douchi extract.

### Animal and AD induction

Male 3-week-old NC/Nga mice (13–15 g each) were obtained from Central Lab Animal Inc. (Seoul, Korea) and maintained in an air-conditioned animal room for 2 weeks before the experiments began. The mice were divided into three groups (*n* = 10 per group) as follows: 1) Ctrl group, which was not induced AD and were given water, 2) AD group, which induced AD and were given water 3) DT group, which Douchi-treated AD-induced mice.

In our experiment, the method used for induction of AD-like skin lesions was depends on previous studies [32–35]. It is as followings. For barrier dysruption, the back regions of the mice was stripped, and 1 mL of 5 % sodium dodecyl sulfate (SDS) (Sigma-Aldrich, St. Louis, MO, USA) was rubbed on the back of each mouse 20 times using a cotton swab to remove the lipid lamella of the stratum corneum. *Dermatophagoides* (*D*.) *farinae* crude extract (100 mg, Biostir Inc., Kobe, Hyogo, Japan) was applied 6 times per week for 3 weeks.

Mice in DT group were orally administered Douchi extract 20 mg/kg for 3 weeks daily. Animals were sacrificed 72 h after the last Douchi administration, and histological specimens were collected. All procedures involving animals were approved by the Institutional Animal Care and Use Committee of Pusan National University (IACUC number: PNU-2014-0732). We followed the NIH Guide for the Care and Use of Laboratory Animals throughout this study. All data were evaluated by observers who were blinded to the experimental conditions.

### Evaluation of skin dermatitis severity

The severity of morphology in the dorsal skin was evaluated after 3 weeks and compared to the baseline. The items of skin score are (1) erythema/hemorrhage (2) scarring/dryness (3) edema (4) excoriation/erosion was scored as 0 (none), 1 (mild), 2 (moderate), or 3 (severe). The sum of the individual scores was defined as the atopy skin score [36].

### Histochemistry

Sodium pentobarbital was used to sedate mice. Anesthetized mice were fixed in 10 % neutral buffered formalin (NBF) solution, and a vascular rinse was performed for 24 h at room temperature. Fixed dorsal tissue slices were embedded in paraffin. After embedding, 5 μm thick sections were histochemically stained.

To investigate general morphology, we performed hematoxylin and Eosin staining. To investigate capillary’s changes such as size and braches, we performed Phloxine-tartrazine staining. To investigate edema changes such as collagen fiber distribution, we performed Masson’s trichrome staining. To investigate the distribution and morphological changes of the mast cells, we performed histochemical staining with Luna’s staining. To investigate the lipid lamella in epithelium, we performed histochemical staining with sudan black B after the samples were prepared according to routine historical procedures for cryo-section.

### Immunohistochemistry

Dorsal skin slices were steeped in proteinase K solution (20 μg/mL) to undergo proteolysis for 5 min. The proteolysed slices were incubated in blocking serum (10 % normal goat serum) for 2 h. Then, the slices were incubated with goat anti-PKC (1:100, Santa Cruz Biotechnology, Inc., Dallas, TX, USA), goat anti-IL4 (1:100, Santa Cruz Biotechnology, Inc., Dallas, TX, USA), goat anti-IL12B (1:100, Santa Cruz Biotechnology, Inc., Dallas, TX, USA), goat anti-p-IκB (1:500, Santa Cruz Biotechnology, Inc., Dallas, TX, USA), goat anti-TNF-α (1:200, Santa Cruz Biotechnology, Inc., Dallas, TX, USA), goat anti-substance P (1:100, Santa Cruz Biotechnology, Inc., Dallas, TX, USA), goat anti-MMP-9 (1:100, Santa Cruz Biotechnology, Inc., Dallas, TX, USA), goat anti-iNOS (1:100, Santa Cruz Biotechnology, Inc., Dallas, TX, USA), all of which are primary antibodies, for 72 h in a 4 °C humidified chamber. Next, the slices were linked with biotinylated rabbit anti-goat IgG (1:100, Santa Cruz Biotechnology, Inc., Dallas, TX, USA), which is a secondary antibody, for 24 h at room temperature. After the slices were exposed to the secondary antibody, an avidin-biotin complex kit (Vector Laboratories, Inc., Burlingame, CA, USA) was applied for 1 h at room temperature. Finally, slices were developed with 0.05 M tris-HCl buffer solution (pH 7.4), which consists of 0.05 % 3,3’-diaminobenzidine and 0.01 % HCl, and then counter-stained with hematoxylin.

**Fig. 2:**
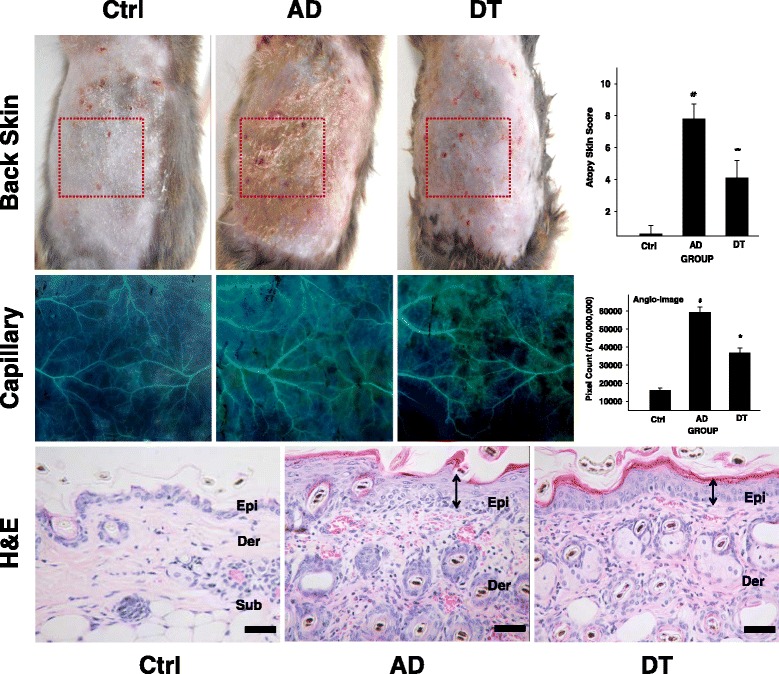
The mitigative effect of Douchi treatment for AD symptoms. The skin features demonstrated visually a marked reduction in DT compared with AD. Incision of red box is analyzed to identify capillary distribution. In our image analysis of the capillaries, the size and branch of capillary increased in the AD, but decreased in the DT (4×). In H&E result, red spot indicates capillary, ↕: hyperplasia of the epithelium. *: *p* < 0.01 compared with AD, #: *p* < 0.01 compared with Ctrl. Bar size: 200 μm (Figure in H&E). Abbreviations; AD: AD-induced mice, Ctrl: non treatment mice, DT: Douchi extract-treated mice after AD inducement, Der: dermis, Epi: epidermis, H&E: Haematoxylin and eosin stain, Sub: subcutaneous layer

We observed skin damage such as elimination of the stratum corneum, epithelial cell hyperplasia, increased infiltration of granular leukocytes and lymphocytes into the basal layer, increased capillary distribution, and increased edema in the dermis. In contrast, the DT group exhibited less skin damage in most regions (Fig. 2).

### Image analysis and statistical analysis

To produce numerical data from our immunohistochemical results, an image analysis was performed using Image Pro Plus (Media Cybernetics, Rockville, MD, USA). Data were presented as the means ± standard error and were evaluated with one-way Analysis of Variance (ANOVA) followed by Duncan’s multiple comparison test among different groups, if needed. The statistical significances of the differences were analyzed with SPSS software (PASW Statistics 18.0, IBM, Armonk, NY, USA) with a significance level of *p* < 0.01.

## Results

### Alleviating effect of skin damage

#### Change of skin features

The repeated application of *D.farinae* crude extract worsened AD-like skin scores and symptoms of hemorrhage, edema, scarring, dryness and erosion. Administering Douchi was observed to alleviate skin damage. The atopy skin score was evaluated 7.8 ± 0.3 in the AD group, 4.1 ± 0.4 in the DT group. Significant differences were shown between all of the groups (*p* < 0.01). In the DT group, clinical scores and symptoms were significantly reduced as compared to AD group (*p* < 0.01) (Fig. 2).

To observe changes in capillaries, we created incisions and pushed aside the skin. Capillaries were photographed with 4× magnification and observed after inversion. In the DT group, the size and number of capillary branches decreased by 38 % compared to the AD group (Fig. 2).

### Regulation of Th2 differentiation

#### Repair of Lipid barrier

Intercellular spaces of epidermal stratum corneum contain extensive arrays lipid lamella, which is mainly composed of ceramide [37]. Ceramide deficiency induced PKC production, that leads to AD-like skin lesion [37].

**Fig. 3:**
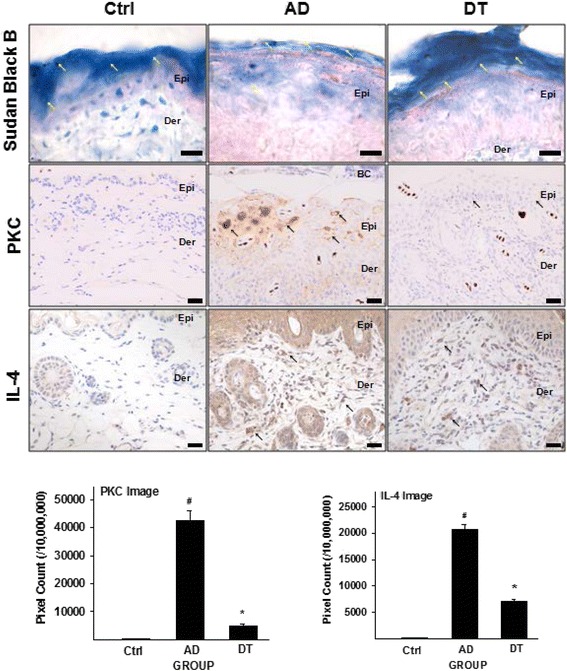
Effects of Douchi on the alleviation of skin barrier dysfunction and Th2 differentiation. In Sudan Black B results, more thicker lipid lamella layer (bright blue) were observed on epidermis in the DT compared with AD. Yellow arrow (↖) indicates lipid lamella. PKC and IL-4 positive reactions (↖, arrow indicates dark brown) were remarkably decreased in DT compared with AD. Data of PKC and IL-4 was also shown same result in graph of pixel count. Bar size: 100 μm. Abbreviation; BC: Blood clot

We identified that layers of lamella were more disappeared in AD group compared with DT group (Fig. 3). The result of Sudan black B staining indicated that the thicker layers of lipid lamella appeared in epidermis of DT group compared with AD group.

#### Amelioration of PKC activity

In the AD group, PKC was activated by ceramide deficiency in stratum corneum by exposure to SDS and *D.farinae*. Immunohistochemical staining with anti-PKC antibodies indicated that a PKC-positive reaction appeared in damaged keratinocytes and in the intercellular space. An increase in the levels of PKC was observed in the AD group compared with the Ctrl group. This elevation was significantly decreased by the oral administration of Douchi. Significant differences were shown between all of the groups (*p* < 0.01). Compared to AD group, PKC was shown to decrease by 88 % in the DT group (*p* < 0.01) (Fig. 3).

#### Regulation of IL-4 production

Increased Th2 cytokine expression is an important feature of AD. An increase in IL-4 production was observed in the AD group compared with the Ctrl group. The results of immunohistochemical staining with anti-IL-4 antibodies indicated the appearance of IL-4-positive reactions in the keratinocytes, lymphocytes and dermal macrophages. Significant differences were shown between all of the groups (*p* < 0.01). Compared with the AD group, Douchi significantly decreased IL-4 levels (*p* < 0.01). The DT group showed a 65 % decrease in IL-4 compared with the AD group (Fig. 3).

### Regulation of inflammation mediator

#### Decrease of mast cell secreted cytokines

Mast cells are tissue-based inflammatory cells. Mast cells are divided into two types as follows: granulated or degranulated. The granulated type is more, but the degranulated type predominates in the inflammatory state. The induced degranulation of mast cells under Th2-dominated conditions plays a key role in inducing inflammatory skin damage [38].

**Fig. 4:**
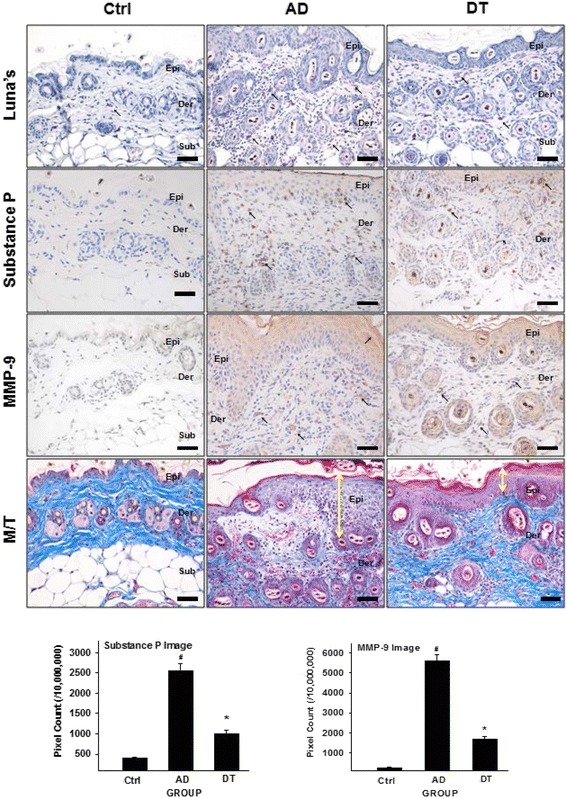
Regulating effects of Douchi on cytokines secretion of mast cell. In Luna’s staining, more granulated mast cells (*arrows indicate purple*) were observed in the dermis of DT than in that of AD. Decreased numbers of substance P-positively reacted cells (*arrow indicates dark brown*) were observed in the DT compared with the AD. In MMP-9 immunohistochemistry results, MMP-9-positively reacted cells (*arrow indicates dark brown in dermal macrophage*) decreased in the DT compared to the AD. In M/T results, collagen fibers (*bright blue*) decreased in AD compared with DT. Arrows (↕) indicate hyperplasia of the epithelium. Bar size: 100 μm. *: *p* < 0.01 compared with AD, #: *p* < 0.01 compared with Ctrl. Abbreviations; M/T : Masson’s trichrome staining

The results of Luna’s staining indicated that many degranulated mast cells appeared from the dermal papilla to the area around the subcutaneous layer in the AD group. On the other hand, fewer mast cells, which were primarily granular, appeared in the DT group compared with the AD group (Fig. 4).

Substance P, which is a neuropeptide related to ache and itching, causes neurogenic inflammation [39]. Substance P level was increased in the AD group. The result of immunohistochemical staining with anti-substance P antibody indicated that substance P-positive reactions appeared in keratinocytes and dermal macrophages. Significant differences were shown between all of the groups (*p* < 0.01). The reactions observed in the DT group decreased by 61 % compared with those observed in the AD group (*p* < 0.01) (Fig. 4).

MMP-9, which is a collagenase, induces tissue destruction by degrading the proteins that constitutes the extracellular matrix and the basement membrane. Consequently, MMP-9 causes edema that facilitates the migration of inflammation-related cells. In the AD group, MMP-9 production increased. The results of immunohistochemical staining with anti-MMP-9 antibody indicated the appearance of MMP-9-positive reactions in keratinocytes and dermal macrophages. Significant differences were shown between all of the groups (*p* < 0.01). The reactions observed in the DT group decreased by 70 % compared with those of the AD group (*p* < 0.01) (Fig. 4).

Repeated *D. farinae* application for 3 weeks induced severe AD-like skin lesions. The result of Masson’s trichrome staining showed decrease of collagen fiber distribution in the AD group 3 weeks after the induction of AD-like skin lesions (Fig. 4). We observed skin damage such as elimination of the stratum corneum, epithelial cell hyperplasia, increased infiltration of granular leukocytes and lymphocytes into the basal layer, increased capillary distribution, and increased edema in the dermis. These are basic histologic patterns of inflammatory skin disease [40]. In contrast, the DT group exhibited less skin damage in most regions (Fig. 4).

#### Regulation of inflammatory cytokine

**Fig. 5:**
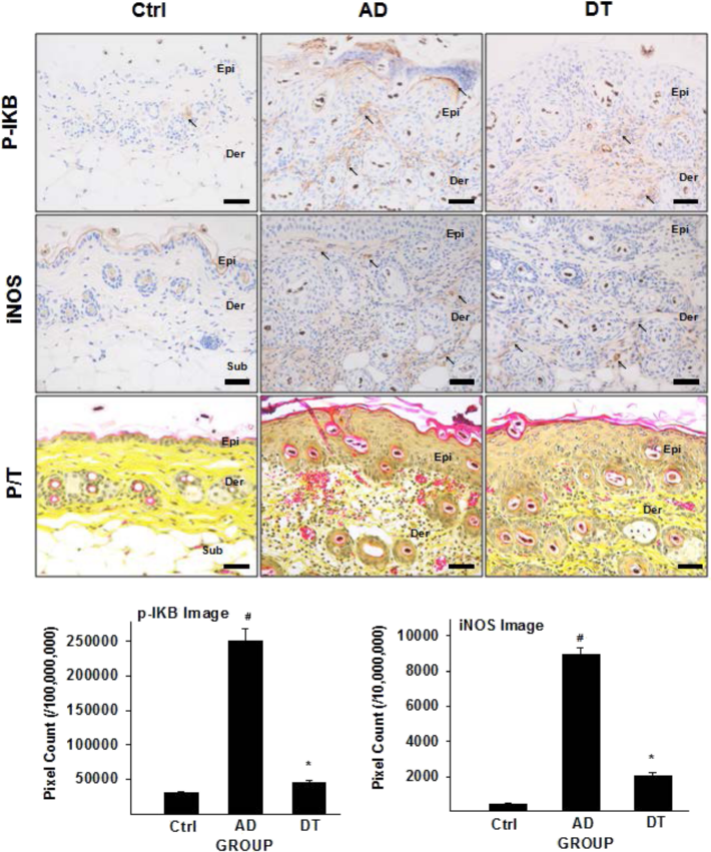
Effects of Douchi on regulation of inflammatory factors (NF-κB and iNOS). P-IκB positive reaction (↖, *arrow indicates dark brown*) decreased in DT compared with AD. iNOS-positively reacted cells (↖, *arrow indicates dark brown*) decreased in DT compared with AD. The capillary size and branches (P/T) were more appeared in the AD compared with DT. Bar size: 200 μm. *: *p* < 0.01 compared with AD, #: *p* < 0.01 compared with Ctrl. Abbreviations; P/T: Phloxine-tartrazine staining

To estimate the Th2 differentiation, we also measured the level of phosphorylated IκB (p-IκB). NF-κB is a transcription factor which plays an important role in expression of Th2 cytokines [41], and the facilitated NF-κB accelerates AD by enhancing the production of inflammatory cytokine [42]. Signaling of p-IκB activates translocation of NF-kB in cytoplasm [43], so we can know production of NF-κB through level of p-IκB. The level of p-IκB increased in lymphocytes and dermal macrophages in the AD group compared with the Ctrl group. The DT group showed a 23 % (*p* < 0.01) decrease in p-IκB as compared with AD group (Fig. 5).

iNOS, produces inflammation by vasodilation, which occurs through the production of nitric oxide, increased in the AD group. The results of immunohistochemical staining with anti-iNOS antibodies indicated the appearance of iNOS-positive reactions in dermal macrophages. Significant differences were shown between all of the groups (*p* < 0.01). The DT group showed a 77 % decrease in iNOS compared with the AD group. There was a significant difference between the AD and Ctrl groups (*p* < 0.01) (Fig. 5). In results of Phloxine-tartrazine staining, the size and branches of capillary were also increased in AD group compared with DT group (Fig. 5).

### Regulation of Th1 differentiation

**Fig. 6:**
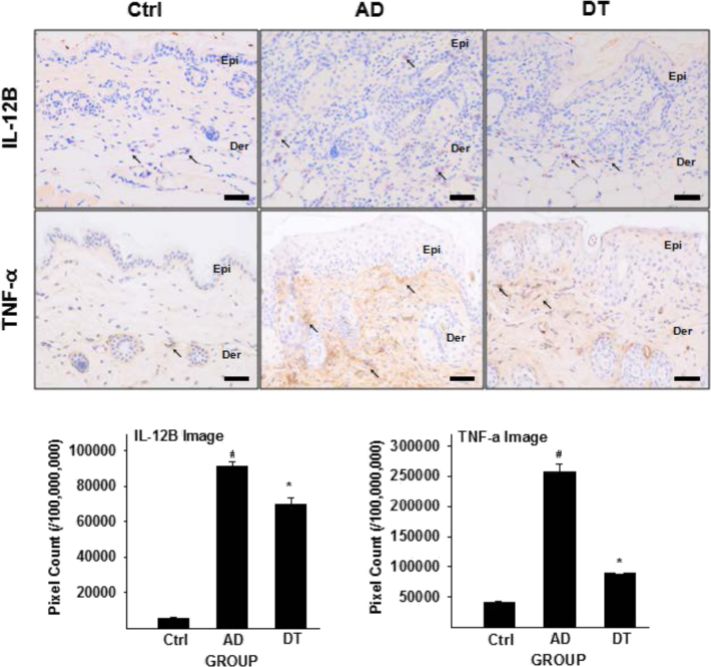
Effects of Douchi on regulation of Th1 differentiation. IL-12 and TNF- α positive reaction (↖, *arrow indicates purple*) decreased in DT compared with AD. Data of IL-12 and TNF- α image analysis were also shown same result in graph of pixel count. Bar size: 100 μm. *: *p* < 0.01 compared with AD, #: *p* < 0.01 compared with Ctrl

To estimate the regulation of Th1 differentiation, we measured the levels of IL-12 and TNF-α positive reaction (Fig. 6). IL-12 induces cell-mediated immunity by up-regulating Th1 cytokines, and TNF-α is responsible for promoting Th1 cell differentiation as pro-inflammatory cytokine in the pathogenesis of chronic inflammatory disease [44]. The levels of IL-12 and TNF-α increased in lymphocyte and dermal macrophages in the AD group compared with the Ctrl group. The DT group showed a 82 % (*p* < 0.01) decrease in IL-12B and TNF-α as compared with the AD group (Fig. 6).

### No side effect

**Fig. 7:**
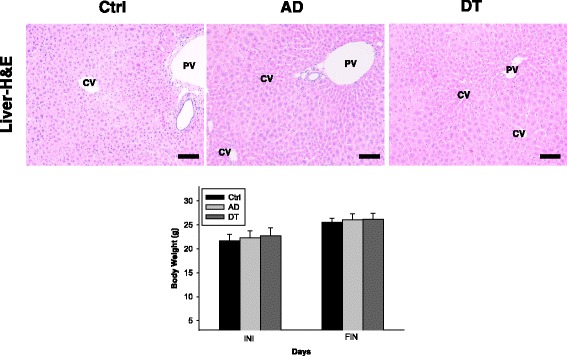
Side effect of Douchi administration. In all groups, no significant damage of liver was observed in H&E result. Significant weight change was also not identified in during the entire experiment in all groups. Bar size: 200 μm. Abbreviations; CV: Central vein, PV: Portal vein, INI: initiation of experiment, FIN: final period of experiment

No significant toxicity such as weight change and liver toxicity was not observed in all groups (Fig. 7). We used oral single dose of 20 mg/kg of Douchi on basis of previous study [45]. Moreover, no significant toxicity was reported in cells or animals treated with soybean related-product [46, 47] and also was reported that Douchi has no toxicity under high-dose up to 2.5 g/kg/day [47].

## Discussion

In this study, we investigated whether Douchi alleviates AD-like skin lesions through modulating PKC and IL-4 in NC/Nga mouse model. We have shown that Douchi decreased the activation of PKC, IL-4 and its downstream targets, mast cells; these effects are associated with decreases in inflammatory mediators such as substance P, MMP-9, and iNOS. Inhibiting inflammatory factors also decreased in AD symptoms such as itching, edema and angiogenesis.

To induce AD-like skin lesion, we used *D.farinae* antigen in mice. *D.farinae* was usually used to induce inflammatory skin lesion in many AD animal experiments [32–35] and which is common house dust mite allergens and known to be suitable method for preparing AD-like skin lesions in experimental animal models [48]. Application of *D.farinae* induced AD-like skin symptoms in mice. In our results, we confirmed morphological skin damage and infiltration of inflammatory cells increased after application of *D.farinae*.

To identify more detailed skin inflammation of all of the groups, clinical severity of the skin lesions was scored by previously described method for human AD [36]. Oral administration of Douchi alleviated clinical sign such as erythema, hemorrhage, edema, erosion, excoriation and scaling. In addition, Douchi also decreased histological changes such as elimination of the stratum corneum, epithelial cell hyperplasia, increased infiltration of granular leukocytes and lymphocytes, increased capillary distribution and decreased collagen fiber distribution (Fig. 2), which are characteristics of inflammatory skin disease.

Now we present our detailed analysis on the mechanism of the AD improvement as follows. Firstly, deficiency of ceramides in stratum corneum is an essential etiologic aspect in the early events of AD [49], where the deficiency of ceramides is represented by PKC level [50]. As shown in Fig. 3, *D.farinae* application decreased both of ceramide in stratum corneum and lipid lamella layers, and increased PKC level. In this regard, we understand that Douchi administration suppressed damage to lipid lamella, which means that Douchi alleviated occurrence of AD through reducing damage to epidermal layers.

Further to damaging the lipid barrier, PKC activates Th2 cell functioning by regulating PKC-dependent Ca^2+^ channels [14]. As shown in Fig. 3, both of PKC and IL-4 were increased in AD group. In contrast, the results showed decreased both PKC level and IL-4 level in DT group. Therefore, it could be understood that PKC affects to IL-4 reduction by suppressing Th2 cell functioning after Douchi administration. The authors believe that Douchi significantly decreased AD-like inflammatory symptoms through regulating IL-4 through suppressing PKC level.

Next, IL-4 promotes release of IgE by B-lymphocyte, and the elevated IgE binds to the high-affinity IgE receptors (FcεRI) on the mast cells and causes the mast cell degranulation [51]. This process plays a key role in the inflammation. From our results such as Figs. 3 and 4, we identified that Douchi suppressed the IL-4 level and the mast cell degranulation. Considering the above relation between the IL-4 promotion and the mast cell degranulation, we understand that our results also show the same results and suppression of mast cell degranulation could be induced by IL-4 suppression.

The mast cell degranulation caused by IL-4 promotion also affects to increase of symptoms of AD such as itching or edema by releasing inflammatory mediators like substance P or MMP-9 [52, 53]. In detail, regarding itch sensation, mast cells are characterized by abundance of secretory granules that contain numerous preformed inflammatory mediators such as histamine, where the secretory granules are bound to itch-related receptor [51]. The bind of secretory granules to the itch-related receptors promotes the release of substance P from the specific sensory nerves. It is known that substance P is one of the most potent pruritogenic peptides [54]. In our results, the mast cell degranulation and the substance P release were observed (Fig. 4). We believe that Fig. 4 showed that Douchi could suppress mast cell degranulation, which led to reduction of the substance P release, considering the above itch sensation mechanism. On the other hand, regarding edema in skin, the mast cell receives stimulatory signals while interacts with activated IL-4, that serves to influence MMP-9 release. MMP-9 induces edema by eliminating collagen fibers in the extracellular matrix, edema is one of characteristics of inflammation [52].

Taken together, we identified the levels of substance P and MMP-9 were significantly suppressed in the DT group when compared with the AD group (Fig. 4). According to these results, the reduction of the substance p and MMP-9 by Douchi administration could contribute to alleviate AD-induced skin edema and morphological changes, suggesting that treatment with Douchi can reveal less inflammatory situations in skin through regulating IL-4.

In addition, the authors believe that there can be other AD pathway beginning from IL-4 to iNOS. Th2 cytokine such as IL-4 activates NF-κB through lipopolysaccharide-induced NF-κB pathway [55], where NF-κB is one of well-known iNOS inducers [56]. Regarding this type of AD pathway, we understand Douchi also shows anti-inflammatory effect as described below. In our study, suppression of IL-4 level was observed, as well as suppression of p-IκB level and iNOS level. Also, our results also showed improvement in AD symptom like angiogenesis. That is, IL-4 suppression could be interpreted as reduction of NF-κB, which causes improvement in suppression of iNOS level, where suppression of iNOS level is considered as mitigation of angiogenesis. In sum, PKC and IL-4 are original effectors inducing AD-like skin lesion, and our results suggests that Douchi reduces infiltration of degranulated mast cells and inflammatory mediators (substance P, MMP-9 and iNOS) through suppression of PKC and IL-4 activity.

To confirm contribution of constituents of Douchi, we confirmed HPLC analysis. Our HPLC analysis revealed Douchi contains isoflavones, such as genistein and daidzein (Fig. 1). The result coincided with previous study that the fermented soybean contains isoflavones [57]. Douchi have more isoflavones including genistein, daidzein, genistin, daidzin than raw soybean (Fig. 1). Previous studies reported that isoflavone inhibits tyrosine kinases, such as PKC [58], and is known to inhibit iNOS [59]. The most constituent of Douchi is genistin, so anti-inflammatory effect of Douchi was considered to be most influenced by genistin. Genistin is known to inhibit effectively pro-inflammatory factors such as TNF-α in inflammatory disease [60]. In addition, genistin and daidzin attenuates collagenase MMP-9 [61]. Therefore, these components were considered to effect alleviate AD-like symptoms through the down-regulation of PKC, iNOS, MMP-9 and TNF-α.

Actually, AD is known to be a biphasic inflammatory skin disorder, provoked by an imbalance between Th1 and Th2 immune responses [44]. So, we identified Th1 cytokine level additionally. To identify Th1/ Th2 imbalance, we measured IL-12 and TNF-α as Th1 markers, IL-4 and p-IκB as Th2 markers additionally. IL-12 and TNF- α plays a key role in Th1 differentiation, while IL-4 and p-IκB is essential for Th2 development. According to our immunohistochemistry result, both of Th1 (IL-12 and TNF- α) and Th2 marker (IL-4 and p-IκB) were decreased after Douchi administration. In addition, damage of lipid lamella resulted in increasing Th1 and Th2 cytokines in our results. Although, Th1/Th2 imbalance is known to be the main characteristic of AD, recently, many studies also reported that up-regulation of Th1 and Th2 cytokines were observed in AD patients [62, 63], and herbal applications decreased Th1 as well as Th2 cytokines, which may lead to alleviation of symptom of AD [64, 65]. Likewise, Douchi also suppressed Th1 and Th2 markers and these results may lead to improvement of AD.

Fermentation is a popular processing method to decrease allergenic property of raw soybean and improve the nutritional properties [66]. By previous study, after the fermentation of soybeans, the contents of the secondary physiologically active ingredients are increased, especially isoflavones and free amino acids [66]. Our results of comparing the components of raw soybean and Douchi are also identified same as previous study. It was shown that Douchi have more isoflavones compared with raw soybean after fermentation. These finding suggests that isoflavones in raw soybean are increased through the process of fermentation, this is considered to affect alleviation of inflammation. Recently, it was reported that *artemisia* have an anti-inflammatory effect in psoriasis animal model [67]. Therefore, in our result of change through fermentation, anti-inflammatory effect of Douchi may be associated with *artemisia* extract. In traditional medicine, Douchi has known to enhance heat-clearing effect in skin through fermentation with *artemisia apiacea* and *mori folium* [21], there are not sufficient evidence at present, so further analysis are needed about contribution of components.

In traditional medicine, the most common pattern of AD is heat, when heat predominates in skin, there may be more redness inflammatory state [17]. Inflammatory diseases such as AD are considered to be heat-abundant states of a body [19]. In a previous study, inflammatory cytokines such as IL-4 were mainly related to the “Hot Zheng (heat-abundant symptoms)” [19]. Thus, traditional medicinal techniques consisting of “purging the heat of the skin” may contribute to suppression of inflammatory cytokines. Douchi has been known in traditional medicine to remove heat and other exterior pathogenic factors due to its ‘cold’ property [21]. In our results, Douchi reduced inflammatory cytokines, including IL-4. Our results suggest that the inhibition of inflammatory cytokines is considered to be a result from one mechanism underlying the ‘heat-clearing’ effects of Douchi.

We identified inhibition of IL-4 in AD-induced NC/Nga mice. In infants with Th1/Th2 imbalance, extrinsic allergen can easily cause Th2-dominant condition that leads to AD. This is our reason for 3-week-old NC/Nga mice, in contrast to be used 7 to 8-week-old NC/Nga mice. These mice are used as a model for children with immune conditions who are sensitive to extrinsic allergens [68]. We confirmed that Douchi downregulated IL-4 and p-IκB (Th2 markers); this demonstrated that Douchi had possibility of the less AD-inducing effect through downregulating Th2 cytokines in infants.

In summary, our results demonstrated that Douchi treatment was effective in attenuating inflammation in AD-induced NC/Nga mice and that this inhibitory effect might be associated with the regulation of the immune response by decreasing Th2 cytokine and PKC activity. Therefore, this study provides evidence that Douchi alleviates inflammation in AD-induced mice and might have potential in the prevention and treatment of AD. However, we believe that further studies are needed to clarify the detailed mechanism of PKC and IL-4 inhibition and the role of Th1 cytokines in the mechanism.

## Conclusion

We attempted to identify inflammation-alleviating effect of Douchi in AD-like NC/Nga mice through inhibiting PKC and IL-4 activation. As these factors are essential factor of AD, these lead to regulate various inflammatory mediators. Therefore, this study provides proof that the oral administration of Douchi inhibits AD-like inflammation in mice and may have potential for the prevention and treatment of AD.

## Abbreviations

AD: Atopic dermatitis
ANOVA: Analysis of Variance
BC: Blood clot
D.: Dermatophagoides
Der: Dermis
Epi: Epidermis
FcεRI: High-affinity IgE receptor I
HPLC: High-performance liquid chromatography
IL: Interleukin
iNOS: Inducible nitric oxide synthase
MMP-9: Matrix metallopeptidase 9
NBF: Neutral buffered formalin
NF-κB: Nuclear factor kappa-light-chain-enhancer of activated B cells
p-IκB: Phosphorylated inhibitor of kappa B
PKC: Protein kinase C
SDS: Sodium dodecyl sulfate
Sub: Subcutaneous layer
Th: T helper
TNF-α: Tumor necrosis factor-alpha
